# Evolutionary sex bias in cognitive response to new environmental risk factor - PM2.5

**DOI:** 10.1186/s13293-025-00774-9

**Published:** 2025-11-05

**Authors:** Hui Chen, Alexei Verkhratsky, Chenju Yi, Brian G. Oliver

**Affiliations:** 1 School of Life Sciences, Faculty of Science, University of Technology Sydney, Ultimo, NSW 2007 Australia; 2 Faculty of Biology, Medicine and Health, The University of Manchester, Manchester, UK; 3 International Joint Research Centre on Purinergic Signalling of Sichuan Province, Chengdu University of Traditional Chinese Medicine , Chengdu, 611137 China; 4 Department of Forensic Analytical Toxicology, School of Forensic Medicine, China Medical University, Shenyang, China; 5 Guangdong Provincial Key Laboratory of Digestive Cancer Research, The Seventh Affiliated Hospital of Sun Yat-sen University, Shenzhen, 518107 China; 6 Department of Geriatrics, Seventh Affiliated Hospital of Sun Yat-sen University, Shenzhen, 518107 China; 7 Shenzhen Key Laboratory of Chinese Medicine Active Substance Screening and Translational Research, Shenzhen, 518107 China

**Keywords:** Air pollution, PM_2.5_, Dementia, Ageing, Alzheimer’s disease, Epigenetic modification, Histone methylation, MicroRNA

## Abstract

The association between exposure to particulates in polluted air and cognitive impairment is an emerging and significant health concern, particularly among younger populations. Although exposure to particulate matter ≤ 2.5 μm (PM_2.5_) is linked with a lower estimated risk for dementia compared to traditional risk factors such as APOEɛ4 gene variants, the widespread and long-term population exposure to PM_2.5_ pose substantial implications for public health. This review explores the sex differences in cognitive function induced by PM_2.5_, which are age-dependent and distinct from the sex bias observed in Alzheimer’s disease. In addition to biological sex and sex hormones, we also discuss the role of epigenetic regulation as a mechanism underlying sex-specific cognitive vulnerabilities to environmental toxins, particularly PM_2.5_. Understanding these differences is important for developing targeted interventions and public health strategies to mitigate the cognitive impacts of PM_2.5_ exposure.

## Overview

The association between exposure to particulate matter (PM) at the microscopic sizes (≤ 2.5 μm in diameter, PM_2.5_) in polluted air and cognitive malfunction is recognised as an area of concern, especially among non-ageing populations [1], yet still mechanistically understudied. This may be due to the fact that the magnitude of effect estimates for the risk of developing dementia linked to air pollution are lower than some traditional ones, such as lifestyle choices, socioeconomic status and genetic predisposition (i.e. APOE ɛ4/4 alleles) [2–5]. Or it may be due to the fact that it’s a relatively recent discovery. However, the population health implications can be significant given that 99% of the world’s population is at risk of exposure to air pollutants. An emerging area of interest is the potential sex differences in changes of cognitive function induced by PM_2.5_, which seems to be age-dependent and can be different from the sex prevalence for Alzheimer’s disease, the leading cause of ageing dementia [6–8]. In addition to the traditional explanation involving sex hormones, epigenetic regulation has emerged as a relevant mechanism.

This paper provides a comprehensive overview of how epigenetic regulation contributes to sex-specific cognitive vulnerabilities in response to environmental toxins, in particular, PM_2.5_. We shall examine key epigenetic mechanisms, discuss the developmental windows of susceptibility, current challenges in studying sex bias, and the implications for public health and future therapeutic interventions.

## Pathophysiology of dementia

Dementia is a gradual decline in cognitive functions (such as memory and learning) and mood control [9]. It is a broad term encompassing various neurodegenerative disorders characterised by a decrease in cognitive function severe enough to interfere with daily life [9, 10].

Dementia is identified as one of the leading causes of disability in later life, contributing to a substantial portion of years lived with disability worldwide. This is because dementia is common, diagnosed in 4% of people over 65 and in 13.1% among those aged 85 and above in the US [11]; whereas an even larger number of patients are never diagnosed. Dementia encompasses a range of disorders with complex aetiologies and pathophysiological mechanisms [12, 13]. The most common form of dementia is Alzheimer’s disease (AD), followed by vascular dementia, frontotemporal dementia, and Lewy body dementia [12]. The aetiology of dementia is multifactorial, involving genetic, environmental, and lifestyle factors. Genetic factors play a significant role in the development of some types of dementia. In familial AD, mutations in genes such as APP, PSEN1, and PSEN2 are linked to early-onset forms of the disease. Additionally, the APOE ε4 allele is a significant risk factor for late-onset AD [14]. Vascular dementia is often associated with genetic predispositions to cardiovascular diseases. Frontotemporal dementia is linked to mutations in the microtubule-associated protein tau (*MAPT*), progranulin (*GRN*), and chromosome 9 open reading frame 72 (*C9orf72*) genes. Although the genetic factors for Lewy body dementia are less well-defined, mutations in the α-synuclein (*SNCA*) and glucocerebrosidase (*GBA*) genes have been implicated [15]. Environmental and lifestyle factors also contribute to the risk of developing dementia. Cardiovascular health is a critical factor, with conditions such as hypertension, diabetes, and hypercholesterolemia significantly increasing the risk of both AD and vascular dementia. Lifestyle choices, including smoking and excessive alcohol consumption, are detrimental and can exacerbate the risk of dementia [16–18]. Conversely, a healthy diet, optimal amount of regular physical activity, quitting smoking and cognitive engagement can reduce dementia risk.

The pathophysiological mechanisms underlying dementia vary by type but often involve common pathways such as neurotoxic protein aggregation, neuroinflammation, and mitochondrial malfunction. In AD, the accumulation of amyloid-β (Aβ) peptides emerges as extracellular plaques that disrupt cell-to-cell communication and trigger immune responses, leading to inflammation and subsequent neuronal damage [19]. Additionally, tau proteins stabilise microtubules, which are essential for maintaining the structure and function of neurons. However, abnormally phosphorylated tau proteins translate into intracellular aggregates of hyperphosphorylated tau protein, leading to the formation of neurofibrillary tangles that accumulate inside neurons and disrupt the microtubule network [19]. This disruption impairs the transport of nutrients and other essential molecules within neurons, ultimately leading to cell death.

Neuroinflammation is another critical aspect of dementia pathophysiology. The brain immune cells assume dystrophic, malfunctional or reactive states in response to amyloid plaques and other pathological changes. Microglia, specialised immune cells of the central nervous system, are key in maintaining brain homeostasis. Microglia accumulate around Aβ plaques [20] in the early stages of AD, to clear toxic substances and slow AD progression [21]. However, as the disease progresses, chronically reactive microglia release pro-inflammatory factors like tumour necrosis factor (TNF)-α and interleukin-1β (IL-1β), favouring an inflammatory environment in the brain, which exacerbates neuronal damage and contributes to neuronal injury and the progression of dementia [22, 23]. Chronic neuroinflammation further exacerbates neuronal damage, creating a vicious cycle of degeneration.

Synaptic malfunction is another hallmark of dementia [24, 25] associated with a loss of synapses and synaptic function, which impairs neurotransmission. This is particularly detrimental to cognitive functions such as memory and learning, which rely on efficient synaptic communications [24, 25].

Mitochondrial malfunction is increasingly recognised as a key contributor to the pathophysiology of dementia, with growing evidence linking impaired mitochondrial function to cognitive decline and neurodegeneration [26, 27]. Mitochondria play a critical role in maintaining neuronal and neuroglial energy homeostasis, regulating oxidative stress, and modulating apoptosis [28, 29]. In dementia, particularly AD, mitochondrial deficits result in reduced ATP production, increased reactive oxygen species (ROS) generation, and impaired mitophagy, leading to the accumulation of aberrant mitochondria [7, 30–32]. These disruptions contribute to synaptic failure, neuroinflammation, and progressive neuronal loss. Furthermore, emerging research suggests that sex-specific differences in mitochondrial dynamics may influence disease susceptibility and progression, highlighting biological sex as a key factor in searching for targeted therapeutic strategies [7, 33, 34].

## New risk factor – PM_2.5_

In the past, the focus on the cause of dementia was on age-related mechanisms; however, dementia has become a health issue among the younger population. In this population, different risk factors appear to be more important than those associated with the risk factors of classical dementia [2]. Chronic exposure to polluted air, especially the particulates i.e., PM_2.5_, is one of the environmental challenges promoting dementia.

### PM exposure and health impact

The human brain is highly susceptible to environmental influences, and increasing evidence suggests that exposure to environmental toxins, such as tobacco smoke and polluted air, can significantly impair cognitive function [3, 16]. Smoking was identified as a negative risk factor for the development of dementia was contingent upon the association of the authors with the tobacco industry, and studies with no association with big tobacco such as a recent cohort study in *JAMA* identified a positive correlation between smoking and the development of dementia [16, 18]. However, smoking rates are at an all-time global low, which has allowed epidemiological studies to identify other, perhaps hidden, risk factors. Air pollution, even in countries assumed to have good air quality, is now emerging as a significant risk factor for almost all smoking-related diseases, such as chronic obstructive pulmonary disease, lung cancer, and dementia [3, 35, 36]. Adverse cognitive function associated with air pollution, ranging from memory deficits to neurodevelopmental disorders, is of particular concern, given the growing prevalence of environmental pollutants globally [3].

PM are small heterogeneous particles suspended in the air that can consist of dust/dirt, soot, smoke, or liquid droplets [37]. PM can come from a variety of man-made and natural sources, including power stations, factories, construction sites, vehicles, fires, as well as dust storms and pollen [38]. The rise in vehicular emissions has turned PM_2.5_ into an ever-looming pressure on humans [37], primarily because of urbanisation and high traffic density and congestion in these areas, resulting in high-level localised exposures. For people living in developed countries traffic related air pollution is the major source of PM_2.5_ exposure. This is unlikely to change for the foreseeable future, as for example even electric vehicles are a significant source. Their greater weights can produce twice the amount of PM_2.5_ from the brakes and tyres than fossil fuel-powered vehicles [39], and without green electricity, electric vehicles may not be the environmental and health panacea that they are perceived to be [40].

PM_2.5_ can penetrate deep into the airways and enter the bloodstream to reach all organ systems, leading to various adverse health effects [41]. It is well established that there is no safe level of air pollution exposure for humans [35, 42]. Australia is often considered to have the best air quality globally. We modelled such exposures, using an Australian level of PM_2.5_ to reproduce local and systemic responses, and found, for example, that sub-chronic and chronic exposure of mice can impair lungs, and instigate diseases of internal organs such as liver steatosis, and cause cognitive impairments [43–45]. Traffic-derived PM_2.5_ exposure raised public concern in Australia, where 11,000 premature deaths and 19,000 hospitalisations in adults per annum and $11–24 billion in health costs resulted from traffic-derived PM_2.5_ [46]. Alarmingly, the general public showed a concerning lack of awareness regarding the dangers of exposure to traffic-related PM. When informed, many people still choose not to take protective measures, such as avoiding polluted areas, and continue to engage in risky behaviours like walking along busy roads [47].

PM exposure affects cognitive function related to learning, memory and social interactions by affecting brain plasticity (the ability to adapt in real time), particularly in the hippocampus, where new information is first received and processed; rather than serving as the final storage site, the hippocampus acts like an index, linking to long-term memories stored elsewhere in the brain, making them accessible ready for retrieval when needed (learning and memory) [48, 49].

### Impact of PM exposure on the risk of dementia

**Fig. 1:**
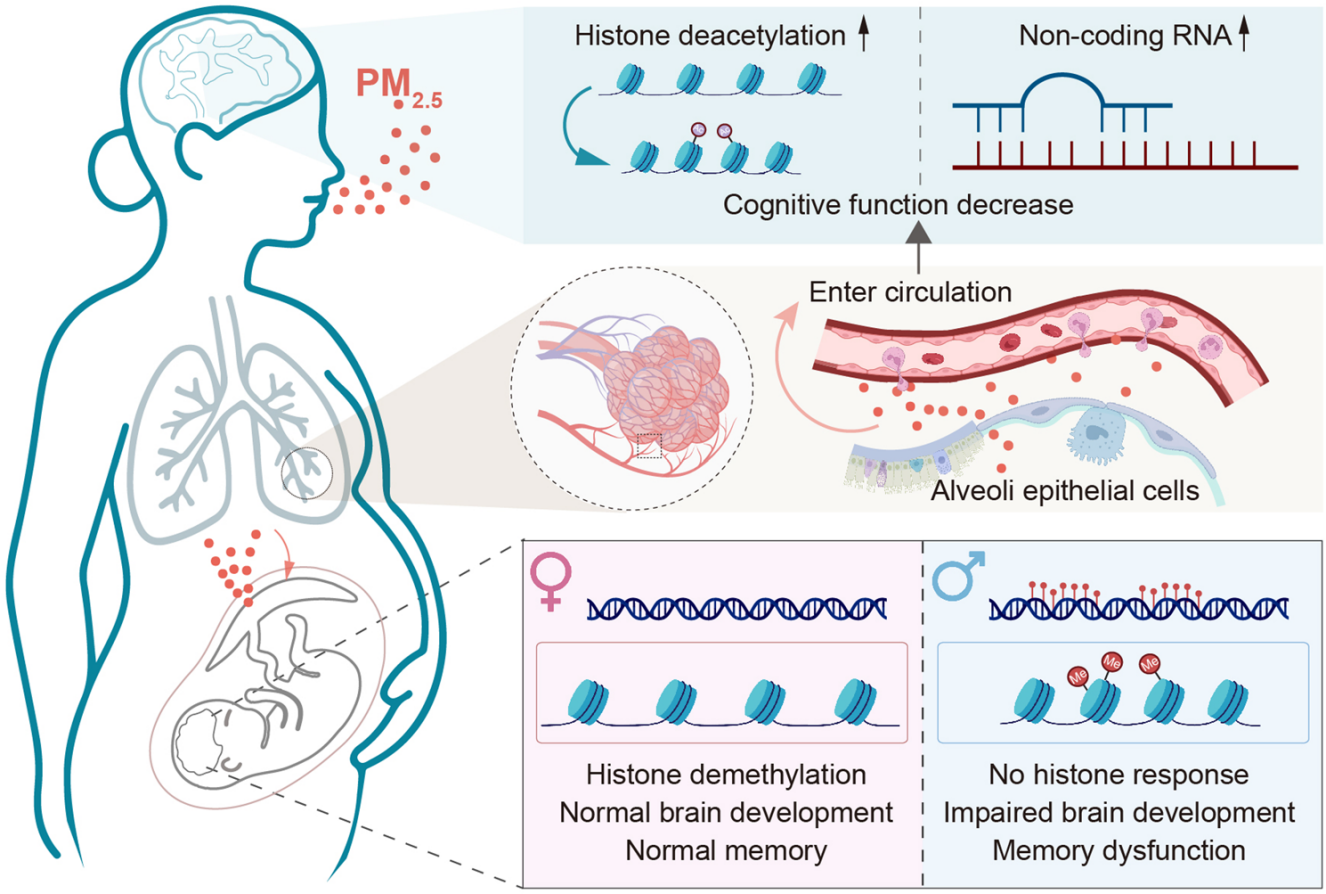
Proposed epigenetic mechanism of PM_2.5_ exposure induced sex differences in cognitive impairment in the first and second generations. Inhaled PM_2.5_ can reach the terminal segment of the lung, the alveoli, where they cross the air-blood barrier to enter the circulation. Due to their small size and potential harmful effects on the blood brain barrier, they will gain access to all cell types in the brain where they induce oxidative stress and inflammatory response, accompanied by altered expression of non-coding RNAs, such as microRNAs and long non-coding RNAs. This can lead to neural injury, accelerate neurodegeneration, and impair cognitive function. Maternal exposure to PM_2.5_ can also lead to epigenetic modifications in the developing foetal brain, including changes in histone methylation and acetylation, particularly involving X- and Y-linked histone demethylases (Kdm5c and Kdm6a). The histone changes are more protected in female offspring, resulting in sex-specific cognitive outcomes. Females show normal brain development during early life, transit memory decline before puberty, but normalised memory function in adulthood; whereas, male littermates exhibit impaired brain development and dysfunctional memory function in adulthood. The interplay between epigenetic regulators and sex hormones during critical developmental windows contributes to differential vulnerability, highlighting the importance of protecting the intrauterine environment from environmental toxins

Cognitive function determines behaviours that are key to individual survival and working ability (e.g., memory and learning) and species extension (e.g., social interactions). Exposure to PM_2.5_ can directly lead to cognitive decline and neurological disorders [37] by gaining access to the brain through two routes. The first involves PM_2.5_ entering the brain from the circulation by crossing the blood-brain barrier, to accumulate in key memory-related regions, such as the prefrontal cortex and hippocampus [38, 50] (Fig. 1). The second route is through the nasal mucosa and olfactory bulb after inhalation, where ultrafine particles can travel along the olfactory nerve into the cerebral cortex and cerebellum [51–53]. The second route, although direct, is not likely to transport the majority of PM_2.5_ into the brain. PM_2.5_ can also affect the brain indirectly. Exposure to ambient PM has been linked to systemic inflammation, predominantly from the lung, disruption of the blood-brain barrier, altered neurotransmitter homeostasis, and an increase in neuroinflammation that may result in neuronal death [37]. A human study showed that grey matter atrophy and memory decline were associated with chronic PM exposure [54].

The link between air pollution levels and dementia is well established in human epidemiological studies [48]. The solid component of polluted air, i.e., PM_2.5_, poses a greater risk than gaseous components [55, 56]. It is estimated that exposure to PM_2.5_ may contribute to ~ 21% of dementia cases, which is significant as a modifiable risk factor [56]. According to a recent study, there is around a 17% increase in the risk of dementia for every 2 µg/m³ rise in average yearly PM_2.5_ exposure [55]. Exposure to PM_2.5_ levels exceeding the US Environmental Protection Agency (EPA) annual standard of 12 µg/m³ is linked to an 81% increased risk of cognitive decline and a 92% higher risk of all-cause dementia, with APOE ɛ4 carriers being particularly vulnerable [57]. Additionally, a 1.5 µg/m³ increase in PM_2.5_ exposure can accelerate the decline in global cognitive function assessed by the Mini-Mental State Examination, including orientation, registration, attention and calculation, recall, language and praxis [58]. PM is not a uniform entity, i.e., it is derived from different sources in different regions. For example, in metropolitan areas of developed countries, vehicle exhausts can be the major contributor to PM pollution, whereas in developing countries, PMs can come from agriculture, industrial and biomass burning. PM_2.5_ from agriculture, traffic and wildfire have a strong association with dementia risk in those above 60 years old, reflecting their high susceptibility [56]. From in vitro research, it is clear that biomass PMs possess greater cellular toxicity than traffic-derived PMs [59]. Although 99% of the world’s population inhales high levels of pollutants, and the health threats are more prominent in low- and mid-income countries [60], most large population epidemiological studies, including meta-analyses of PM and cognitive decline, were performed in the US and Europe [55]. As such, some studies narrowed their focus on the risk of traffic-related PMs. Exposure to high levels of traffic-related PM_2.5_ is also linked to increased cognitive decline; however, it does not necessarily cause brain imaging changes [54], suggesting potential attribution of cellular functional changes. Again, the gaseous component in traffic pollution did not seem to affect cognition [61].

The hazard ratio of PM_2.5_ exposure for clinical dementia often varies depending on the research method used to measure or estimate exposure, and may therefore reflect inherent limitations or potential underestimation. For example, most studies on PM rely on residential history and environmental data derived from air quality monitoring and simulated atmospheric chemistry [56]. In human studies, a spatial resolution of 1 km is typically used to calculate exposure and correlated to health effects obtained from medical records [48]. According to a meta-analysis, for every 2 µg/m^3^ PM_2.5_, the total hazard ratio was 1.04 (95% confidence interval 0.99 to 1.09), which suggests a small effect [55]. However, when “active case ascertainment” was used which is a proactive method used in public health research to identify cases of a disease or condition through direct outreach and systematic screening, rather than relying on existing records or passive reporting, the hazard ratio was increased to 1.42 (1.00–2.02) [55]. This suggests the need to closely examine the research methodology when interpreting risk estimates.

Dementia is increasingly recognised not only as an ageing condition, but also as a significant health issue affecting younger populations. Young people living in heavily polluted areas show impairment in intelligence, memory, and school performance [49]. In recent years, young-age onset memory decline and dementia are on the increase in insurance claims [62]. Data from the UK Biobank identified PM as a modifiable risk factor for young-onset dementia. Even though PM levels were only slightly elevated in the incident young-onset dementia group (10.31versus 10.01 µg/m^3^), PM exposure had a hazard ratio of 1.25 & 1.26 for young-onset dementia in 2 statistical models tested [2]. The real-life risk of dementia was likely to be more than the number reported due to reliance on hospital records and limited areas with PM surveillance [63]. While the incidence of young-onset dementia is lower compared to late-onset forms, its impact can be disproportionately severe. Individuals in their working years face unique challenges, as they bear the responsibility of earning income, supporting aging parents, and maintaining social interactions. This dual burden can lead to heightened personal, societal, and economic consequences. Addressing air quality is important in mitigating risks and improving outcomes for younger individuals affected by this condition.

### Sex differences in response to ambient PM

The adverse impacts of PM_2.5_ are heterogeneous across populations. There is a pronounced sex bias in cognitive responses to PM_2.5_ exposure, with males and females often exhibiting different susceptibility and outcomes at different ages.

AD has a strong female prevalence [64]. Similar sex bias has also been found in research on PM exposure; however, the effects are not as pronounced. For example, Mini-Mental State Examination among 1,484 Koreans aged 55 and older showed a slightly higher risk of cognitive impairment among women than men (OR 1.03) with increased exposure to PM_2.5–10.5_ (ranging from 13.86 to 22.23 µg/m^3^), although the average score of the Mini-Mental State Examination was similar between women and men [65]. However, the PM exposure level of women was significantly higher, while their education levels were significantly lower than those of men [65], which may skew the higher risk towards women. Nevertheless, most epidemiological studies on PM often focus on aged women [66]. Chronic exposure to PM_2.5_ was linked to AD-like cognitive impairments among females [54], where PM_2.5_ exposure is associated with greater declines in immediate recall and new learning with no impact on delayed recall or overall cognitive scores [54]. Specifically, for each 2.81 µg/m³ interquartile rise in PM_2.5_, the annual rate of decline accelerated by 14.8–19.3% after controlling for confounders [54]. In another cohort of women (71–81 years old), an increase in 10 µg/m³ of PM exposure over an extended period of time is linked to cognitive deterioration comparable to ageing by about two years [66]. Moreover, older women residing in areas with outdoor PM_2.5_ levels exceeding the US Environmental Protection Agency (EPA) threshold were nearly twice as likely to develop clinically significant cognitive impairment compared to those living in areas with lower ambient PM_2.5_ levels [57]. Over a five-year period, older women living in areas with elevated PM_2.5_ levels faced a 24% higher risk of developing dementia compared to those in low-exposure areas; they were also more likely to exhibit an AD-like phenotype, including brain grey matter atrophy [67]. In brain regions commonly affected by AD, greater exposure to air pollutants is associated with reduced cortical thickness [68]. The decline in episodic memory observed in these older women is likely attributed to such brain morphological changes.

However, the focus on elderly females only can induce a selection bias on both age and sex. Indeed, a study in Shanghai during a period when the average daily ambient PM_2.5_ level was 55 µg/m^3^ showed that the risk of non-accidental deaths was smaller in women than in men [69]. Men also tended to die younger than women (4.4 years difference). Inclusion of four age groups: < 40, 40–50, 60–79, and ≥ 80 years, identified the low risk in women among those < 40 years of age. Younger individuals are probably more vulnerable to PM_2.5_ induced impact, and the cognitive outcomes that can affect life-long learning and working ability, as well as mental health status [70].

Children of 6–14 years of age dwelling in an area with high indoor PM levels due to coal fly ash from nearby coal-fired power plants showed predominant cognitive impairment in females [71]. Among three measurement criteria (Behavior Assessment and Research System, Continuous Performance Test and Selective Attention Test), girls performed poorly in the Continuous Performance Test (incidence rate ratio = 1.39, 95% CI = 1.06, 1.82), suggesting a high risk of impulse control problems, whereas boys showed normal performances throughout [71]. One special aspect of that study is that it analysed responses before and during puberty. Significant physiological changes during puberty due to hormonal remodelling may also affect synaptic plasticity and brain wiring, making cognitive performance not only more dynamic but also distinct from that of adulthood, when neurodevelopmental changes have largely stabilised. Indeed, in an animal model, in-utero PM_2.5_ exposure due to maternal exposure leads to a temporary memory impairment in pre-puberty females, which was corrected in adulthood [7]. Evolution favours female survival and longevity to maintain species survival. When the *in-utero* environment is challenged by inhaled toxins, the brain health of the male foetus shows significant cellular stress and dysfunction, which can last until adulthood, whereas their female littermates seem to be protected from these in-utero exposures [7, 72–74]. Similarly, pollutants have a stronger negative effect on male cognitive performance, specifically verbal and math skills, than females living in the same environment, more so in those older and less educated participants [6]. The advantage of this study [6] is that it covers a large population aged 10 years old and above. It seems to have limitations on racial background by only focusing on residents in China; however, it is still representative of cumulative exposure effects. In addition, the predominance of adverse effects due to long-term PM_2.5_ exposure in males is similar to a mouse model of a low exposure level of traffic-derived PM_2.5_ [7], which excluded all confounding factors characteristic for human studies.

The above research findings suggest that the sex-dependent response to PM-induced cognitive impairment may also be age-dependent. In the mouse model where PM_2.5_ was studied alone, females showed some impairment of spatial memory function before puberty, which was normalised during adulthood [7]. It is unclear if such re-adjustment also occurs in humans, as studies have not so far had a similar follow-up.

## Biological basis for sex differences

### Biological sex

Sex plays a significant role in shaping brain development and cognition including memory [75]. Variations in the brain anatomy and physiology between males and females are evident across multiple species, including humans, and result from a combination of genetic, hormonal, and environmental influences. Men tend to have greater volumes of the amygdala and thalamus than women, whereas females generally exhibit a larger hippocampus compared to males. Additionally, the amygdala contains a higher density of androgen receptors, while the hippocampus is characterised by a greater abundance of estrogen receptors [76]. These differences go beyond physical structure, impacting brain function, which may determine the sex preference for certain neurological disorders, such as AD [77].

As an environmental toxin, PM_2.5_ can impact brain structures which may be drivers of sex differences in cognitive responses [6]. White matter comprises myelinated axons connecting different regions of grey matter, thus creating the brain connectome [78]; critical for high-level cognitive functions, such as verbal skills, planning, and executive functions [6, 79]. A longitudinal study [79] assessed the impact of PM_2.5_ below the US Environmental Protection Authority (EPA) standard levels on the development of white matter microstructure in children aged nine to thirteen, utilising restriction spectrum imaging. It included 8,182 participants across 21 US urban areas, all under 10 years old at the start. One year of PM_2.5_ exposure was associated with microstructure change in the white matter, such as increased isotropic diffusion assessed by MRI at age nine, which may reflect an accelerated brain maturation, which has been associated with impaired cognitive and emotional development [79]. Furthermore, the pollutants influenced the progression of white matter maturation between 9 and 13 years of age, with a broader impact on females [79]. Regarding restriction spectrum imaging, PM_2.5_ influenced a greater number of brain tracts in females, with minimal impact on males [79]. These tracts connect brain regions involved in the planning and execution of complex and goal-oriented behaviours, and disruption in the development of these pathways during adolescence may negatively affect learning, executive and emotional functions. Although our review focuses on PM_2.5_, other gas components (i.e. N_2_ and O_3_) in polluted air also affect white matter development in a sex-dependent manner [79], which makes the changes in white matter development even more complex to interpret. Furthermore, exposure to PM_2.5_ at levels below EPA recommendations led to more structural damage in white matter compared with grey matter, including a decrease in myelin, reduced axonal fibre density, and/or less directional axonal fibre coherence [79, 80]. In males, PM_2.5_ exposure-induced white matter damage results in impaired verbal function [6], while in females, track damage was apparent in adolescence without linking to any specific cognitive dysfunction [79], which may suggest an unknown adaptive mechanism in the female brain to preserve cognitive function. White matter tract damage is being increasingly recognised as a significant histopathological feature of AD, in particular, the disruption of oligodendrocyte integrity related demyelination and axon loss [81, 82]. It can also predict the development of neurocognitive disorders (including AD) in aging populations when it forms part of white matter hyperintensities [83, 84]. Furthermore, the burden of white matter abnormalities can also accelerate amyloid accumulation and cognitive decline in those with AD risk factors, such as APOE ε4 genotype [83, 84]. Importantly, recent research highlights that older females are more vulnerable to the effects of white matter tract damage than males [85]. In addition, risk factors, such as type 2 diabetes, seem to worsen white matter damage in women, which may also increase their susceptibility to dementia [85]. With the prolonged life span, especially in women, this early life abnormality in white matter microstructure may still increase the susceptibility to the detrimental cognitive outcomes during ageing or in the presence of an additional risk factor.

Therefore, PM_2.5_ can time-dependently suppress white matter integrity even at the levels deemed to be safe [80], with more prominent cognitive functional impairment in males. Although no significant cognitive dysfunction was exhibited in females during childhood and adolescence, longitudinal studies are needed to determine whether puberty can reverse the adverse effects of PM_2.5_.

### Sex hormones as a driver of susceptibility to dementia

Sex hormones are processed differently by neurons in various parts of the brain, and they can act independently or synergistically to affect cognitive behaviours, including stress responses, learning, and memory. The influence of sex hormones on cognitive function starts from neuronal development and persists throughout the lifespan [76]. Estrogen supplementation is associated with enhanced verbal skills, which may explain why women generally tend to excel in verbal cognition tasks, such as fluency, vocabulary, verbal memory, and verbal learning [86, 87]. On the other hand, androgens have been linked to improved performance in mathematical and visuospatial tasks, areas where men often show an advantage [88, 89]. Emerging evidence suggests that PM_2.5_ exposure can accelerate the decline of circulating oestradiol during the menopausal transition which is a period now recognised as a critical window during which hormonal changes significantly increase dementia risk in women [8, 90]. Whether PM_2.5_-induced hormonal changes contribute to the doubled likelihood of developing AD observed in ageing women remains to be determined and warrants further investigation [8].

Prenatal androgen exposure enhances spatial abilities and social behaviours, whereas this response decreases during puberty [76]. However, rodent research suggests that sex differences induced by sex hormones, particularly testosterone, are only critical within a narrow window - embryonic day 18 to postnatal day 10 (equivalent to humans gestational days >80 to weeks 36–40 [91, 92], when they induce lasting sex-specific changes in brain structure and function [93]. The mechanisms by which *in utero* exposure to PM_2.5_ influences sex hormone-driven brain development in early life and subsequent formation of memory and cognitive functions are not fully understood, which represents a significant knowledge gap.

### Epigenetic regulation

Since the industrial revolution, human-induced environmental changes intensified, which requires rapid genomic adaptation to avoid extinction. However, changing DNA sequence requires thousands, if not millions of years, to evolve even with stable environmental factors. Alternatively, epigenetic modifications offer a faster genetic response. This is because epigenetic modification does not require changes in gene coding but promotes or suppresses the production of functional proteins to change cellular and subsequently systemic physiology to better withstand environmental stressors that might otherwise be lethal [94]. These epigenetic modifications, serving as a “master switch”, are expected to have long-lasting effects on brain function to enable efficient adaptation, even into the following generations. Epigenetics is often mediated through mechanisms such as DNA methylation (made directly to DNA), histone modifications (made to the amino-acid residues in histone tails), and non-coding RNAs. The effects of DNA methylation, involving the addition of a methyl group to the DNA molecule, typically at cytosine bases in CpG islands on animal behaviour, particularly within the hippocampus, are well understood and reviewed [95–98]. However, the other epigenetic mechanisms involved are poorly understood, which are focused here to raise attention (Fig. 1).

#### Histone modifications

Histone modifications, such as acetylation and methylation, play a key role in regulating chromatin structure and gene expression. In dementia, specific histone modifications have been discovered. For example, Euchromatic Histone Lysine Methyltransferase 1 and 2 encode the enzymes G9a and GLP, which are responsible for the methylation of histone H3 at lysine 9 (H3K9) to silence genes in AD [99]. Histone deacetylases (HDACs) remove acetyl groups from histones, leading to a more condensed chromatin structure to prevent gene expression. Inhibitors of HDACs are being developed as potential therapeutic agents for AD [99]. Insights into the sex difference in epigenetic response are still developing and primarily derived from basic research, including studies using cultured cells and animal models, yet the understanding remains poor due to the preference for using males in most publications.

The X and Y chromosomes are frequently perceived merely as the sex chromosomes, a misconception rooted in the textbook descriptions and a general lack of awareness about X- and Y-encoded genes. Most genes on the Y chromosome have counterparts on the X chromosome, known as X-Y pairs. Contrary to the common assumption, genes existing as X-Y pairs do not necessarily have identical functions, as these genes exhibit up to 90% sequence homology [100]. This highlights the nuanced and specialised roles that these gene variants play in cellular processes. Notably, we were the first to report X-Y-chromosome-linked histone-related brain adaptations predominantly mediated by neuronal Kdm5c and Kdm6a in response to in-utero PM_2.5_ exposure [7]. Kdm5c and Kdm6a are lysine specific histone demethylases that unlock chromatin to promote gene transcription [101]. Kdm5c plays a critical role in fine-tuning enhancer activity during the process of neuronal maturation, and mutations in Kdm5c contribute to X-linked neurodevelopmental disorders [102, 103] when the knockout mice exhibit a range of abnormalities, including cognitive impairments, deficits in social behaviour, memory problems, increased aggression, and a heightened susceptibility to seizures [103, 104]. Deletion of Kdm6a in mice resulted in distinct defects in brain development in males compared to females [105], bolstering a sexually dimorphic role for KDM6A in brain development. Kdm6a deficiency in the hippocampus has been linked to a reduction in the number of dendritic spines, thus impairing the formation of new memories. In addition, KDM6C can regulate the inflammatory response of macrophages [106], which is highly relevant to microglial response to PM_2.5_ exposure and perhaps related risk of synapse loss, memory impairment, and neurodegenerations.

Kdm5c and Kdm6a are only responsive for in-utero PM_2.5_ exposure in female brains, contributing to their differential cognitive outcomes from their male littermates after birth, where females displayed short-term memory malfunction only before puberty, whereas males showed both short-term and learning working dysfunction after puberty [7]. This may be linked to unique patterns of X-chromosome inactivation escape [75]. Particularly, Kdm5c in the pseudo-autosomal region (meaning its Y-chromosome allele has the same coding) was only increased in females by in-utero PM_2.5_ exposure. The functional significance was further backed up by upregulating Kdm5c or Kdm6a in foetal primary neurons in vitro, effectively mitigating PM_2.5_-induced oxidative stress and neural death. Furthermore, in vitro study also suggested the redundancy of Kdm5c and Kdm6a when downregulating one did not diminish the foetal neuronal survival [7]. Clearly, they also have “backups” which was supported by the observation that suppressing both did not show additive toxic effects on the foetal neurons [7]. However, as sex-dependent memory function outcomes are closely related to puberty, there may be an interplay between these two chromosome-linked demethylases and sex hormones, which remains a knowledge gap. This needs to be followed up in future studies (Fig. 1).

#### Non-coding RNAs

Non-coding RNAs, including microRNAs (miRNAs) and long non-coding RNAs (lncRNAs), regulate gene expression at the transcriptional and post-transcriptional levels. These non-coding RNAs can modulate the stability and translation of mRNAs, affecting the protein synthesis key for memory formation and retrieval. In dementia, several non-coding RNAs were identified as key regulators (Fig. 1). For example, miR-29 targets genes involved in Aβ production and tau phosphorylation. Dysregulation of miR-29 has been linked to increased Aβ levels and tau pathology in AD [107]. Reduced levels of miR-132 observed in AD, are involved in synaptic plasticity and neuronal survival [107]. β-Secretase 1 (BACE1), also known as the β-site amyloid precursor protein cleaving enzyme 1, is a key enzyme in the formation of Aβ, operational in the pathogenesis of AD [108]. The long non-coding BACE1 antisense RNA (BACE1-AS) regulates BACE1 expression. Elevated levels of BACE1-AS have been associated with increased Aβ levels in AD [107]. PM_2.5_ exposure has been shown to increase brain BACE1 in mice, which correlates with suppressed miR-574-5p in the hippocampus [109].

In the hippocampus of rats exposed to PM_2.5_, 21 upregulated and 46 downregulated lncRNAs were identified. These lncRNAs are involved in neurodevelopment, memory, and cognitive behaviour, as well as in the MAPK signalling pathway, glutamatergic and cholinergic synapse signalling pathways, and the Foxo signalling pathway [110]. In addition, 11 upregulated and 17 downregulated miRNAs were also found in these rats, which are involved in PI3K/Akt signalling pathway, axon guidance, and the MAPK signalling pathway [110]. Network analysis suggests that IncRNA-miRNA target genes are involved in the synaptic vesicle cycle, the Ras signalling pathway, and the MAPK signalling pathway [110]. In particular, the upregulation of miR-770-3p in the hippocampus directly inhibits the expression of Sox10 (known as sex-determining region Y-box 10) [110]. Sox10 promotes chromatin unwinding to facilitate the transcription of genes involved in myelination, synapse formation, and cognitive function [110].

During in-utero PM_2.5_ exposure, foetal brains can be affected by PM_2.5_ crossing the blood-placental barrier, as well as chemokines and cytokines coming from the maternal lung response. As a result, the brain reactions can differ significantly from those induced by direct PM_2.5_ inhalation. An increased miR-6315, miR-3588, and miR-466b-5p expression was found in the cortex and miR-3560 and let-7b-5p in the hippocampus of the foetus [111]. Reduced miR-338-5p and let-7e-5p (which regulate the genes involved in neuroinflammation and sleep) were found in the cortex, whereas miR-99b-5p, miR-92b-5p, and miR-99a-5p were reduced in the hippocampus which have been predicted to inhibit learning abilities [111]. In addition, increased cortical expression miR-30a-3p was detected, which is implicated in younger-onset dementia [111–113]. In another study, in-utero PM2.5 exposure inhibited several microRNAs in the foetal hippocampus, including rno-miR-443-3p, rno-miR-151-3p, rno-miR-107-3p, rno-miR-181a-5p, rno-miR-139-5p, which are predicted to be involved in the pathogenesis of AD [114]. Indeed, the offspring showed impaired long-term memory and working memory [114].

However, whether there are any sex differences in the aforementioned miRNAs and lncRNA response induced by PM_2.5_ exposure remains unknown, which represents another critical knowledge gap.

## Future perspectives

Sex differences in the brain structure and function align with the susceptibility to dementia. The increasing understanding of the interplay between environmental toxins and genetic response requires significant attention. Unique protein isoforms produced by Y-encoded genes, while essential for specific cell and tissue functions, may pose disadvantages in terms of epigenetic adaptation to environmental challenges. These isoforms can limit the flexibility of gene expression responses, making it harder for cells to adapt to changing environmental conditions. Such rigidity can result in a reduced ability to mitigate damage from environmental stressors, potentially leading to increased susceptibility to diseases or disorders linked to environmental factors. Additionally, the specialised nature of these isoforms might hinder the overall genomic plasticity required for effective epigenetic regulation and adaptation. However, it also needs to be noted that the adaptive advantage in the female brain is not without the cost of other organ(s). For example, to cope with a suboptimal in-utero environment, the vital organs, including the brain, receive priority allocation of nutrients at the cost of the other, such as the liver. We have found in the same female mice with in-utero PM_2.5_ exposure, although their memory functions are normal in adulthood, their liver changes are suggestive of fatty liver like pathologies, including increased lipid accumulation accompanied by increased inflammatory response and fibrotic accumulation [115]. Regarding the sex biases, preserving cognitive function in females can make them more resilient and survive longer within a suboptimal environment when the species is threatened; whereas, male’s contribution to reproduction can be accomplished within a short life span. This is also reflected by the increased female offspring number in the litter [44]. This can maximise the reproductive capacity.

Epigenetic regulation of gene expression is a highly dynamic process, and recent research has highlighted that these mechanisms do not act in isolation but are part of a complex regulatory network with extensive crosstalk, which, for example, is increasingly recognised as a key factor in mediating cancer development [99]. Histone modifications alter chromatin structure and thereby regulate gene accessibility and transcription. Non-coding RNA further modulates gene expression at both transcriptional and post-transcriptional levels. Notably, non-coding RNAs can recruit chromatin-modifying complexes to specific genomic loci, thereby influencing histone modification patterns and chromatin state [116]. There is a bidirectional crosstalk between histone modifications and non-coding RNAs [116]. For example, certain histone marks (such as H3K36me3) can guide the deposition of RNA modifications like N6-methyladenosine (m6A) on nascent transcripts, which in turn can regulate the recruitment of chromatin-modifying enzymes to specific genomic regions [117]. Conversely, non-coding RNAs can act as scaffolds or guides for histone-modifying complexes, influencing the local chromatin environment and gene expression programs critical for neuronal plasticity and cognitive function [117]. Sex differences in cognitive responses may arise from sex-specific patterns of histone modifications and non-coding RNA expression, influenced by hormonal milieu and genetic background. Estrogen-responsive non-coding RNAs have been shown to interact with histone-modifying enzymes, leading to sex-dependent epigenetic landscapes in the brain [117–119]. These differences can affect synaptic plasticity, memory formation, and vulnerability to neuropsychiatric disorders [119]. However, how this crosstalk responds to direct or in-utero PM_2.5_ exposure is unclear, nor is the sex difference, which leaves a critical knowledge gap. Understanding these knowledge gaps is not only fundamental to advancing our knowledge of brain development and function but also essential for developing sex-specific targeted interventions and treatments. Understanding these knowledge gaps is not only fundamental to advancing our knowledge of brain development and function but also essential for developing sex-specific targeted interventions and treatments.

It is imperative to integrate these insights into public health policies and clinical practices to better address the unique needs of both sexes in the face of environmental challenges. The critical window of the in-utero when environmental toxins exposed by the mothers can exert a life-long impact on the future risk of dementia also highlights the critical importance of protecting the intrauterine environment to mitigate the risk of lifelong neurological issues in the offspring.

## Conclusion

The natural evolution of sex differences in brain structure and function is a critical area of study that has profound implications for our understanding of brain disorders and the impact of environmental toxins. As research continues to unravel the complexities of this topic, it is imperative that we consider sex as a significant biological variable that shapes individual responses to both internal and external environmental challenges. Understanding these differences is important, as it can inform targeted interventions and public health strategies.

## Data Availability

No datasets were generated or analysed during the current study.
